# Single and Combinative Impacts of Healthy Eating Behavior and Physical Activity on COVID-19-like Symptoms among Outpatients: A Multi-Hospital and Health Center Survey

**DOI:** 10.3390/nu13093258

**Published:** 2021-09-18

**Authors:** Minh H. Nguyen, Thu T. M. Pham, Dinh N. Vu, Binh N. Do, Hoang C. Nguyen, Thai H. Duong, Khue M. Pham, Linh V. Pham, Thao T. P. Nguyen, Cuong Q. Tran, Quyen H. Nguyen, Thanh M. Hoang, Khanh V. Tran, Trang T. Duong, Shwu-Huey Yang, Chyi-Huey Bai, Tuyen Van Duong

**Affiliations:** 1 International Ph.D. Program in Medicine, College of Medicine, Taipei Medical University, Taipei 110-31, Taiwan; d142108015@tmu.edu.tw; 2 Faculty of Public Health, Hai Phong University of Medicine and Pharmacy, Hai Phong 042-12, Vietnam; phamminhthu.ytcc@gmail.com (T.T.M.P.); pmkhue@hpmu.edu.vn (K.M.P.); 3 School of Public Health, College of Public Health, Taipei Medical University, Taipei 110-31, Taiwan; 4 Director Office, Military Hospital 103, Hanoi 121-08, Vietnam; vunhatdinh@vmmu.edu.vn; 5 Department of Trauma and Orthopedic Surgery, Vietnam Military Medical University, Hanoi 121-08, Vietnam; 6 Department of Infectious Diseases, Vietnam Military Medical University, Hanoi 121-08, Vietnam; nhubinh.do@vmmu.edu.vn; 7 Division of Military Science, Military Hospital 103, Hanoi 121-08, Vietnam; 8 Director Office, Thai Nguyen National Hospital, Thai Nguyen City 241-24, Vietnam; nguyenconghoang@tnmc.edu.vn (H.C.N.); dhthaivn@gmail.com (T.H.D.); 9 President Office, Thai Nguyen University of Medicine and Pharmacy, Thai Nguyen City 241-17, Vietnam; 10 Department of Internal Medicine, Thai Nguyen University of Medicine and Pharmacy, Thai Nguyen City 241-17, Vietnam; 11 President Office, Hai Phong University of Medicine and Pharmacy, Hai Phong 042-12, Vietnam; 12 Department of Pulmonary & Cardiovascular Diseases, Hai Phong University of Medicine and Pharmacy Hospital, Hai Phong 042-12, Vietnam; pvlinh@hpmu.edu.vn; 13 Director Office, Hai Phong University of Medicine and Pharmacy Hospital, Hai Phong 042-12, Vietnam; 14 Health Management Training Institute, University of Medicine and Pharmacy, Hue University, Thua Thien Hue 491-20, Vietnam; nguyenthiphuongthao@hueuni.edu.vn; 15 Director Office, Thu Duc City Health Center, Ho Chi Minh City 713-10, Vietnam; quoccuong.mph@gmail.com; 16 Faculty of Health, Mekong University, Vinh Long 852-16, Vietnam; 17 Department of Anesthesiology, Thu Duc City Hospital, Ho Chi Minh City 713-11, Vietnam; huuquyenhm@gmail.com (Q.H.N.); hoangminhthanhbvct@gmail.com (T.M.H.); 18 Director Office, Le Van Thinh Hospital (Previously Hospital District 2), Ho Chi Minh City 711-13, Vietnam; bskhanh70@yahoo.com.vn; 19 Nursing Office, Tan Phu District Hospital, Ho Chi Minh City 720-16, Vietnam; duongtrang7273@gmail.com; 20 School of Nutrition and Health Sciences, Taipei Medical University, Taipei 110-31, Taiwan; sherry@tmu.edu.tw; 21 Research Center of Geriatric Nutrition, Taipei Medical University, Taipei 110-31, Taiwan; 22 Nutrition Research Center, Taipei Medical University Hospital, Taipei 110-31, Taiwan; 23 Department of Public Health, College of Medicine, Taipei Medical University, Taipei 110-31, Taiwan

**Keywords:** COVID-19-like symptoms, healthy eating score, fruits, vegetables, fish, physical activity, non-pharmacological, interaction, immune function, Vietnam

## Abstract

Background: Healthy eating and physical activity are effective non-pharmacological approaches to boost immune function and contain the pandemic. We aimed to explore the associations and interactions between physical activity and healthy eating behavior with COVID-19-like symptoms (Slike-CV19S). Methods: A cross-sectional study was conducted on 3947 outpatients, from 14 February to 2 March 2020, at nine health facilities in Vietnam. Data collection included sociodemographic characteristics, healthy eating behavior (using the healthy eating score (HES) questionnaire), physical activity (using the short form international physical activity questionnaire), and Slike-CV19S. The associations and interactions were tested using logistic regression models. Results: Frequent intake of fruits (OR = 0.84; *p* = 0.016), vegetables (OR = 0.72; *p* = 0.036), and fish (OR = 0.43; *p* < 0.001) were associated with a lower Slike-CV19S likelihood, as compared with infrequent intake. Patients with higher HES levels (OR = 0.84; *p* = 0.033 for medium HES; OR = 0.77; *p* = 0.006 for high HES) or being physically active (OR = 0.69; *p* < 0.001) had a lower Slike-CV19S likelihood, as compared to those with low HES or physical inactivity, respectively. Patients with medium HES who were physically active (OR = 0.69; *p* = 0.005), or with high HES and physically active (OR = 0.58; *p* < 0.001), had a lower Slike-CV19S likelihood, as compared to those with low HES and physical inactivity. Conclusions: Healthy eating behavior and physical activity showed single and combinative impacts on protecting people from Slike-CV19S. Strategic approaches are encouraged to improve healthy behaviors, which may further contribute to containing the pandemic.

## 1. Introduction

Deaths due to COVID-19 have exceeded 3 million globally, as reported in June 2021 [1]. Although COVID-19 vaccination rollouts have been implemented worldwide at different rates [2,3], the emergence of new variants and waning immunity after vaccination or infection over time have prevented the community from achieving herd immunity [4,5]. Consequently, new waves of the COVID-19 disease have re-emerged [6,7], which have negatively impacted healthcare systems and socio-economies in many countries [8,9,10].

COVID-19 is a contagious respiratory illness with symptoms similar to other respiratory infections (e.g., influenza) [11]. Suspected COVID-19 symptoms (Slike-CV19S) adversely affect people’s physical health and increase fear and nervousness, even if people are not confirmed with COVID-19 infection. In addition, Slike-CV19S have been linked to an elevated risk of mental illnesses and poorer quality of life [12,13]. Moreover, individuals with any suspected symptom were advised to stay at home, self-isolate, or keep a safe distance from others [14]. This leads to feelings of loneliness and isolation, which may exacerbate people’s psychological ill-health. Moreover, people with Slike-CV19S have to go to medical facilities for COVID-19 testing, thereby causing financial burden and pressure on the health system [15,16].

During the pandemic, the general public seeking medical care were at a higher risk of infection [17]. They have encountered many stressors, including limited access to healthcare services, delayed treatments, fear of contracting COVID-19, and health concerns [17,18,19]. In particular, previous research showed that patients with underlying diseases and Slike-CV19S had a higher likelihood of experiencing mental health problems [20]. Therefore, investigating protective factors is highly recommended to protect patients from Slike-CV19S.

A diverse and healthy diet could help to mitigate the risk of chronic diseases [21,22] and improve health outcomes [23]. Frequent consumption of healthy foods (e.g., vegetables, fruits, fish) helps to provide the body with a sufficient intake of essential nutrients and antioxidants [24,25], which could reduce inflammation and oxidative stress, enhancing immunity [26,27]. In addition, physical activity could help to lower the respiratory infection likelihood, as reported in previous studies [28,29]. Physical exercise can help to strengthen immune function [30,31]. Therefore, healthy eating and staying physically active could mitigate the susceptibility to pathogens that helps to decrease the chance of infectious respiratory diseases, including COVID-19. 

In a similar situation to other countries, Vietnam has been facing significant challenges in containing the coronavirus infection and managing its consequences. Several preventive measures have been implemented, such as social distancing, quarantine, and lockdown, which may negatively affect lifestyles (e.g., increase in sedentary behavior, unhealthy eating) [32,33]. Thus, it is essential to provide timely evidence about the beneficial roles of physical activity and healthy diets in health, encouraging patients to engage in these behaviors to stay healthy during the crisis.

Therefore, the aims of this study were to explore the single and combinative impacts of healthy eating behavior and physical activity on the likelihood of Slike-CV19S among outpatients.

## 2. Materials and Methods

### 2.1. Study Design

An observational cross-sectional study was conducted on outpatients from 14 February to 2 March 2020. Patients were recruited during their visit to the outpatient departments (OPDs) of nine health centers and hospitals in Vietnam. 

This study was reviewed and approved by all selected health facilities and the ethical committee of Hanoi University of Public Health (IRB number: 029/2020/YTCC-HD3). 

### 2.2. Study Population and Data Collection Procedure

The study participants were recruited using the consecutive convenience sampling technique. Inclusion criteria were people aged 18–85, voluntarily participating in the survey, and without Vietnamese language barriers. Patients experiencing any medical emergencies were excluded (e.g., positive with COVID-19, heart attack, severe injuries, appendicitis, severe bleeding, etc.). Patients unable to communicate normally (e.g., blindness or deafness) were also excluded. 

The process of collecting data was described in a previous publication [12]. In brief, after receiving training in data collection, investigators (physicians, nurses, medical students) contacted and invited outpatients to participate in the survey voluntarily. Patients signed informed consent forms prior to completing this survey. Investigators and participants were required to adhere to precaution measures (e.g., handwashing, mask-wearing, physical distancing) to prevent virus infection during data collection. Each interview took about 20 min. Participant information was obtained and kept confidential and for scientific purposes only. 

A total of 4029 individuals agreed to participate and complete the survey [12]. Out of the sample, 82 respondents were excluded (aged <18 or >85, or missing data). Finally, we analyzed the data of 3947 outpatients.

### 2.3. Measurements

#### 2.3.1. COVID-19-like Symptoms

At the time of the survey, patients were screened for suspected COVID-19 symptoms (Slike-CV19S), including those related to the bronchial system (cough, dyspnea, sputum production, sore throat, rhinorrhea), gastrointestinal system (diarrhea and vomiting), and others (fever, myalgia, fatigue, confusion, headache, chest pain, hemoptysis) [34]. Patients who had any of these symptoms were classified as having Slike-CV19S but did not test positive for COVID-19.

#### 2.3.2. Healthy Eating Behaviors

We used a Healthy Eating Score questionnaire with five items (HES-5) to assess the participant’s healthy eating behavior. The HES-5 was validated and used in the Vietnamese context, with satisfactory validity [35]. The questionnaire investigated patients regarding the frequency of consumption of five healthy foods over the previous 30 days, including vegetables, fruits, dairy products, whole grains, and fish. The frequency of intake of each food was recorded with six possible responses from 0 = “Rarely or never” to 5 = “3 or more times per day”. According to the American Heart Association’s healthy dietary guidelines [36], we categorized the frequency of each healthy food into two groups (frequent vs. infrequent) as follows: “<3 times/day” vs. “≥3 times/day” for vegetables, dairy products, and whole grains; “<2 times/day” vs. “≥2 times/day” for fruits. It is also recommended that adults should consume 1–3 servings of fish per week depending on the type of fish [37]. Thus, for ease of analysis, fish consumption was later categorized into two groups: <1 time/week vs. ≥1 time/week.

Next, the Healthy Eating Score (HES) was calculated by summing up participants’ responses to five items. The total score ranges from 0 to 25, in which patients with a greater score had a better healthy eating behavior. Then, we categorized healthy eating scores into tertiles, a method used previously [38], and defined the first tertile (HES < 10), the second tertile (10 ≤ HES < 15), and the third tertile (HES ≥ 15) as low, medium, and high HES, respectively.

#### 2.3.3. Physical Activity

Physical activity (PA) was evaluated using the short form of the International Physical Activity Questionnaire (IPAQ-SF), which has been utilized for surveys in different countries [39,40,41]. The IPAQ-SF was also validated and used for study in Vietnam [12,42], with acceptable reliability and validity [43,44]. The IPAQ-SF comprises seven items asking participants about the amount of time spent on a variety of PA intensities, from sitting to vigorous activities, during the previous seven days. The PA intensity was expressed as the Metabolic Equivalent Task in minutes/week scores (MET-min/week). The total MET-min/week was the sum of minutes spent on walking, moderate, and vigorous activities multiplied by 3.3, 4.0, and 8.0, respectively. Following IPAQ guidelines [45], MET-min/week was classified into three levels (low, moderate, and high). We then categorized PA into two groups: inactive (low) vs. active (moderate/high) [46].

#### 2.3.4. Covariates

Data on participants’ sociodemographic characteristics were obtained, including age, gender, marital status (single vs. married vs. separated/divorced/widowed), education degree (junior high school and below vs. senior high school vs. college/university/postgraduate degree), occupational status (no job vs. having a job), the ability to pay for healthcare (difficult vs. easy), social status (low vs. middle or high). The information regarding participants’ lifestyle behaviors was collected: smoking (no vs. yes) and alcohol consumption (no vs. yes). Body Mass Index (BMI, kg/m^2^) was calculated and classified into three groups (underweight (BMI < 18.5), normal weight (18.5 ≤ BMI < 25.0), and overweight/obese (BMI ≥ 25.0)). Underlying diseases were assessed using items of the Charlson Comorbidity Index [47] and divided into two groups (no vs. yes).

Health literacy was evaluated using the short version of the Health Literacy Questionnaire (HLS-SF12), which was validated and used in Vietnam [12,48,49,50] and Asian countries [51,52]. The HLS-SF12 comprises twelve items evaluating the perception of difficulty when participants conduct each item, with a 4-level response from “very difficult” (1) to “very easy” (4). The scoring formula is described in previous studies [53]. The total score ranges from 0 to 50, in which patients with greater scores had higher health literacy levels.

### 2.4. Statistical Analysis

Studied variables were expressed as mean, standard deviation, frequency, and percentage. The proportion of Slike-CV19S in different groups of studied variables was tested using a chi-square test or one-way ANOVA test, appropriately. Then, the associations of food intake frequency, healthy eating score, and physical activity with Slike-CV19S were examined using simple and multiple logistic regression analyses. Age, gender, and factors associated with Slike-CV19S at *p*-value < 0.2 in simple logistic regression models (Table S1 in Supplementary File) were adjusted in multiple logistic regression models. For the multicollinearity check, Spearman’s correlation was conducted to check correlations of adjusting factors (Table S2 in Supplementary File). If a moderate correlation (rho ≥ 0.3) was found between two variables, we chose a representative variable to adjust in the multiple logistic regression models. Finally, multiplicative interaction models were used to explore the combinative impact of PA and healthy eating scores on suspected COVID-19 symptoms. We ran the interaction model with three terms (X1, X2, X1 * X2) and potential confounders, where X1 is physical activity (treated as the binary variable: inactive vs. active), X2 is the healthy eating score (treated as the ordinal variable: low, medium, high), X1 * X2 is the interaction terms (including physical activity * medium HES or physical activity * high HES). Odds ratio (OR) with a 95% confidence interval (95% CI) and *p*-value were presented. The *p*-value < 0.05 was defined as a significant level. Data were analyzed using STATA for Windows, version 15.1 (StataCorp LLC, College Station, TX, USA).

## 3. Results

### 3.1. Participant Characteristics Stratified by Suspected COVID-19 Symptoms

A total of 3947 outpatients were analyzed. According to the Vietnam Ministry of Health report, there was no COVID-19 infection at the studied locations during the survey period [54]. Therefore, no outpatients with Slike-CV19S were COVID-19 positive. The mean age of patients was 44.4 years. Of all outpatients, 35.1% (1387/3947) had suspected COVID-19 symptoms, 28.1% were physically inactive, and 72.6% had medium or high HES. Furthermore, 55.7% were women, 23.5% were aged 60–85 years, 41.6% had a college or university degree or higher, 54.9% had a job, and 15.6% had one or more pre-existing diseases. Regarding food intake frequency, fish (85.3%) was most frequently consumed, followed by fruits (55.4%), whole grains (15.4%), dairy products (7.2%), and vegetables (6.3%) (Table 1).

### 3.2. Associations of Food Frequency, Healthy Eating Score, and Physical Activity with COVID-19-like Symptoms

After adjusting for potential confounders, Table 2 shows that frequent vegetable consumption (≥3 times/day) (odds ratio, OR, 0.72; 95% confidence interval, 95% CI, 0.52, 0.98; *p* = 0.036), frequent fruit consumption (≥2 times/day) (OR, 0.84; 95% CI, 0.73, 0.97); *p* = 0.016), and frequent fish consumption (≥1 time/week) (OR, 0.43; 95% CI, 0.36, 0.52); *p* < 0.001) were associated with lower odds of experiencing suspected COVID-19 symptoms. As compared to people with low healthy eating scores (HES), those with medium HES (OR, 0.84; 95% CI, 0.71, 0.98; *p* = 0.033) or high HES (OR, 0.77; 95% CI, 0.64, 0.93; *p* = 0.006) had lower odds of having Slike-CV19S. In comparison to physically inactive people, those with a high level of physical activity had a lower likelihood of having Slike-CV19S (OR, 0.69; 95% CI, 0.59, 0.80); *p* < 0.001).

### 3.3. Interactions between Physical Activity and Healthy Eating Behavior

Table 3 shows the combinative impacts of HES and PA on COVID-19-like symptoms. After adjusting for confounders, there are negative multiplicative interactions between high HES and PA (OR, 0.58; 95% CI, 0.39, 0.86; *p* = 0.008). In other words, in people with high HES, physical activity reduces the likelihood of having Slike-CV19S more than in people with low HES. Regarding the combined effect, Table 3 shows that as compared to people with low HES and physical inactivity, those with medium HES and a high level of physical activity had a 31% lower likelihood of having Slike-CV19S (OR, 0.69; 95% CI, 0.54, 0.89; *p* = 0.005); those with high HES and a high level of physical activity had a 42% lower likelihood of having Slike-CV19S (OR, 0.58; 95% CI, 0.44, 0.77; *p* < 0.001) (Table 3).

## 4. Discussion

In this study, no participants with Slike-CV19S were COVID-19 positive, as no new COVID-19 case was reported at the studied sites during the survey period [54]. However, finding factors associated with the likelihood of COVID-19-like symptoms may provide timely evidence for effective interventions that help to raise people’s awareness and promote health throughout the pandemic.

Our findings show that people who frequently consumed vegetables or fruits were less likely to have COVID-19-like symptoms than those who did not. Like other respiratory infections (e.g., influenza), the symptoms of COVID-19 are manifestations of airway and systemic inflammation [55]. Vegetables and fruits contain a wide range of essential vitamins, antioxidants (e.g., vitamin A, C, E), and bioactive substances (e.g., flavonoids) [56], which can mitigate inflammation, thereby supporting the immune system to fight against viral and bacterial infections, including COVID-19 [27,57,58,59,60]. In addition, adequate vegetable and fruit intake was associated with a lower likelihood of wheezing and chronic inflammatory illnesses (e.g., asthma) [25]. Previous studies indicated that regularly consuming fruits and vegetables could help to lower the likelihood of non-communicable diseases (e.g., COPD, diabetes, cardiovascular diseases) [24,61,62], which may in turn decrease susceptibility to opportunistic infections [63]. We also found that people who regularly ate fish were less like to have suspected symptoms of COVID-19. Fish intake provides the body with omega 3, vitamin D, and selenium [57]. These nutrients have anti-inflammatory effects that may help to mitigate the risk of non-communicable diseases and combat infections [27,64,65]. Particularly, omega-3 fatty acids were found to be linked to better lung function [66], and reduced likelihoods of chronic cough and wheeze [67,68] in previous studies. Therefore, the frequent consumption of fruits, vegetables, and fish has potential health benefits in improving immune responses, which in turn decrease the development of respiratory infection symptoms, including COVID-19. 

In the current study, patients with a medium or high healthy eating score (HES) had lower odds of having Slike-CV19S than those with a low HES. The consumption of healthy and diverse foods helps the body achieve optimal nutrient intake, enhancing the immune response that prevents it from becoming infected with pathogens such as influenza or coronavirus [27,57]. Other healthy diets (e.g., Mediterranean or Dietary Approach to Stop Hypertension diets) were identified by the high intake of vegetables, fruits, whole grains, and fish, which are anti-inflammatory foods [69,70]. In addition, healthy diets were linked to a decreased risk of respiratory illnesses (e.g., COPD, asthma, wheezing symptoms) [71,72,73,74,75]. Adherence to a well-balanced diet was also found to be linked to a decreased likelihood of obesity [76] and chronic health conditions [77,78], which helps decrease the vulnerability to viral infections. Therefore, healthy eating habits have potential health-promoting impacts that help to reduce inflammation, and improve the immune function to protect the body against respiratory infection symptoms. 

Our results indicate that active PA was associated with lower odds of having Slike-CV19S. Prior studies showed that medium and high PA levels were negatively correlated to the likelihood of contracting upper respiratory tract infections [28,29,79,80]. Physical activity has been shown to have anti-inflammatory effects and to lower inflammation, thereby enhancing immune function, which could protect the body against pathogens and reduce the symptoms of respiratory infections [30,31,81,82,83]. In addition, staying physically active was found to be associated with the reduced likelihood of obesity and chronic diseases in previous studies [77,84,85]. These illnesses have been linked to a constant status of low-grade inflammation and immunological dysfunction, which provide greater susceptibility to infectious diseases, including COVID-19 [30]. Therefore, regular engagement in physical activity has potential preventive impacts on the likelihood and symptoms of respiratory infections, and should be encouraged in the time of the pandemic.

Importantly, we found that the combinative impact of PA and a healthy diet was better at reducing the likelihood of suspected symptoms of COVID-19. Our findings suggested that patients with active PA and a more healthy diet could reduce the odds of COVID-19-like symptoms by up to 42% compared to those with inactive PA and low healthy eating. People have faced significant changes in their lives throughout the pandemic, including fear, sleep disorders, and lifestyle changes (e.g., unhealthy eating and inactive PA) [32]. These factors may adversely affect physical and mental health, leading to the functional impairment of immunity [86,87], increasing sensitivity to opportunistic infections, including COVID-19. Therefore, among COVID-19 prevention strategies, the combination of healthy eating and PA should be promoted to reinforce immunological health, reducing the likelihood of suspected symptoms of COVID-19. 

The study has the limitations of a cross-sectional design and reporting bias. Healthy eating was assessed using the HES-5 questionnaire, which includes only five food items and does not query portion sizes. This questionnaire is not suitable for assessing overall dietary intake and may lead to subjective dietary assessments. However, the HES-5 is brief, simple, and well correlated with the 2015 Healthy Eating Index [88]. Therefore, it is a useful tool for quickly assessing diet quality during the sensitive time of the pandemic. This study did not investigate the consumption of unhealthy foods and culture-related aspects, which may potentially affect our findings. In addition, we have not investigated whether patients received nutritional guidance, which may potentially influence patients’ behaviors and affect the analysis. Future research should take these considerations into account in assessments. Moreover, we investigated specific symptoms instead of diseases that caused those symptoms. However, COVID-19-like symptoms (in populations who were negative for COVID-19) play important roles in reflecting immune response. The roles of nutrition and physical activity can be potentially assessed. Furthermore, patients with underlying illnesses may change their diet and exercise regimen according to the suggestions of healthcare professionals. This may also potentially confound the associations. However, we have checked the associations of underlying diseases with healthy eating behavior and PA and found no significant association in this study. The findings are presented in Table S3 in the Supplementary File. Finally, The IPAQ-SF is prone to overestimating PA levels compared to other objective measures (e.g., accelerometer), but it is a valid and commonly used tool to assess PA [40]. However, the strength of the study is its relatively large sample size. Thus, our findings could provide timely and valuable evidence for public health interventions to lower the likelihood of suspected symptoms of COVID-19.

## 5. Conclusions

The single and combinative impacts of physical activity and healthy eating behavior were found to protect patients from having suspected COVID-19 symptoms. Therefore, these healthy behaviors should be promoted to improve patients’ health and well-being. This further contributes to control of the pandemic.

## Figures and Tables

**Table 1:** Characteristics of participants (*N* = 3947).

Variables	Total (*n* = 3947)	Without Slike-CV19S (*n* = 2560)	With Slike-CV19S (*n* = 1387)	
	*n* (%)	*n* (%)	*n* (%)	*p* *
Age (years), mean ± SD	44.4 ± 17.0			
Age groups				<0.001
18–59	3019 (76.5)	2080 (68.9)	939 (31.1)	
60–85	928 (23.5)	480 (51.7)	448 (48.3)	
Gender				0.762
Female	2197 (55.7)	1430 (65.1)	767 (34.9)	
Male	1747 (44.3)	1129 (64.6)	618 (35.4)	
Marital status				<0.001
Single	865 (22.0)	653 (75.5)	212 (24.5)	
Married	2850 (72.4)	1795 (63.0)	1055 (37.0)	
Separated/Divorced/Widowed	220 (5.6)	105 (47.7)	115 (52.3)	
Education attainment				<0.001
Junior high school and below	1216 (30.9)	717 (59.0)	499 (41.0)	
Senior high school	1083 (27.5)	754 (69.7)	328 (30.3)	
College/university/postgraduate degree	1639 (41.6)	1081 (66.0)	558 (34.0)	
Occupational status				0.010
No job	1770 (45.1)	1185 (66.9)	585 (33.1)	
Having a job	2155 (54.9)	1358 (63.0)	797 (37.0)	
Ability to pay for healthcare				<0.001
Very or fairly difficult	1764 (44.7)	964 (54.6)	800 (45.4)	
Very or fairly easy	2182 (55.3)	1595 (73.1)	587 (26.9)	
Social status				<0.001
Low	482 (12.2)	265 (55.0)	217 (45.0)	
Middle/High	3464 (87.8)	2294 (66.2)	1170 (33.8)	
BMI				<0.001
Underweight	386 (9.8)	225 (58.3)	161 (41.7)	
Normal weight	3128 (79.4)	2012 (64.3)	1116 (35.7)	
Overweight or obese	428 (10.9)	320 (74.8)	108 (25.2)	
Underlying diseases				0.936
No	3309(84.4)	2139 (64.6)	1170 (35.4)	
Yes	611 (15.6)	396 (64.8)	215 (35.2)	
Smoking				0.580
No	3465 (88.0)	2252 (65.0)	1213 (35.0)	
Yes	471 (12.0)	300 (63.7)	171 (36.3)	
Drinking alcohol				0.140
No	2653 (67.8)	1702 (64.2)	951 (35.8)	
Yes	1262 (32.2)	840 (66.6)	422 (33.4)	
Physical activity				<0.001
Inactive	1111 (28.1)	628 (56.5)	483 (43.5)	
Active	2836 (71.9)	1932 (68.1)	904 (31.9)	
Food intake frequency				
Vegetables				<0.001
<3 times/day	3697 (93.7)	2372 (64.2)	1325 (35.8)	
≥3 times/day	249 (6.3)	188 (75.5)	61 (24.5)	
Fruits				<0.001
<2 times/day	1761 (44.6)	1111 (63.1)	650 (36.9)	
≥2 times/day	2185 (55.4)	1449 (66.3)	736 (33.7)	
Whole grains				0.098
<3 times/day	3338 (84.6)	2184 (65.4)	1154 (34.6)	
≥3 times/day	607 (15.4)	376 (61.9)	231 (38.1)	
Dairy				0.906
<3 times/day	3659 (92.8)	2375 (64.9)	1284 (35.1)	
≥3 times/day	285 (7.2)	184 (64.6)	101 (35.4)	
Fish				<0.001
<1 time/week	581 (14.7)	259 (44.6)	322 (55.4)	
≥1 time/week	3363 (85.3)	2301 (68.4)	1062 (31.6)	
Healthy Eating Score (HES), mean ± SD	12.0 ± 4.2			<0.001
Low (HES < 10)	1082 (27.4)	649 (60.0)	433 (40.0)	
Medium (10 ≤ HES < 15)	1806 (45.8)	1201 (66.5)	605 (33.5)	
High (HES ≥ 15)	1054 (26.7)	709 (67.3)	345 (32.7)	
Health literacy (HL), mean ± SD	29.9 ± 7.7	30.9 ± 7.7	28.0 ± 7.4	<0.001

Abbreviations: Slike-CV19S, COVID-19-like symptoms. * The *p*-value of the chi-square or one-way ANOVA test appropriately.

**Table 2:** Associations of food frequency, healthy eating score and physical activity with COVID-19-like symptoms (*n* = 3947).

Variables *	COVID-19-like Symptoms			
	Unadjusted Model		Adjusted Model **	
	OR (95% CI)	*p*	OR (95% CI)	*p*
Food intake frequency				
Vegetables				
<3 times/day	1.00		1.00	
≥3 times/day	0.58 (0.43, 0.78)	<0.001	0.72 (0.52, 0.98)	0.036
Fruits				
<2 times/day	1.00		1.00	
≥2 times/day	0.87 (0.76, 0.99)	0.035	0.84 (0.73, 0.97)	0.016
Whole grains				
<3 times/day	1.00		1.00	
≥3 times/day	1.16 (0.97, 1.39)	0.098	0.97 (0.81, 1.18)	0.793
Dairy				
<3 times/day	1.00		1.00	
≥3 times/day	1.01 (0.79, 1.30)	0.906	1.03 (0.79, 1.34)	0.819
Fish				
<1 time/week	1.00		1.00	
≥1 time/week	0.37 (0.31, 0.44)	<0.001	0.43 (0.36, 0.52)	<0.001
Healthy Eating Score (HES)				
Low (HES < 10)	1.00		1.00	
Medium (10 ≤ HES < 15)	0.75 (0.64, 0.88)	<0.001	0.84 (0.71, 0.98)	0.033
High (HES ≥ 15)	0.73 (0.61, 0.87)	<0.001	0.77 (0.64, 0.93)	0.006
Physical activity				
Inactive	1.00		1.00	
Active	0.61 (0.52, 0.70)	<0.001	0.69 (0.59, 0.80)	<0.001

* All variables were tested separately in different models. ** Adjusted for age, gender, occupational status, education attainment, medication payment ability, social status, BMI.

**Table 3:** Interaction between physical activity and healthy eating score on COVID-19-like symptoms (*n* = 3947).

Healthy Eating Score (HES)	Physical Activity				Multiplicative Interaction	
	Inactive		Active			
	OR (95% CI)	*p*	OR (95% CI)	*p*	OR (95% CI)	*p*
Low (HES < 10)	1.00 *		0.88 (0.67, 1.16)	0.380		
Medium (10 ≤ HES < 15)	0.99 (0.74, 1.33)	0.979	0.69 (0.54, 0.89)	0.005	0.79 (0.55, 1.12)	0.182 **
High (HES ≥ 15)	1.13 (0.81, 1.58)	0.458	0.58 (0.44, 0.77)	<0.001	0.58 (0.39, 0.86)	0.008 ***

Abbreviations: HES, healthy eating score. ORs are adjusted for age, gender, occupational status, education attainment, medication payment ability, social status, BMI. * The single reference group is “physical inactivity and low HES”. ** The *p*-value of the interaction term: medium HES * physical activity. *** The *p*-value of the interaction term: high HES * physical activity.

## Data Availability

Data will be available on the reasonable request from the corresponding author.

## Supplementary Materials

The following are available online at https://www.mdpi.com/article/10.3390/nu13093258/s1; Table S1: Confounders associated with suspected COVID-19 symptoms among outpatients (*n* = 3947), Table S2: Spearman’s correlation (rho) among potential confounders (*n* = 3947), Table S3: Associations of underlying diseases with healthy eating behavior and physical activity among outpatients (*n* = 3947).
