# Supplementary material for: Single and Combinative Impacts of Healthy Eating Behavior and Physical Activity on COVID-19-like Symptoms among Outpatients: A Multi-Hospital and Health Center Survey
Source: Nutrients. 2021 Sep 18;13(9):3258. doi: 10.3390/nu13093258 (PMC8465237; doi:10.3390/nu13093258)
Supplement: Supplementary file 1 [file nutrients-13-03258-s001.zip › nutrients-1332353-SI.pdf]

Single and Combinative Impacts of Healthy Eating Behavior and Physical Activity on Symptoms like COVID-19 among Outpatients: A Multi-Hospital and Health Center Survey

Supplemental Material

Table S1. Confounders associated with suspected COVID-19 symptoms among outpatients (n= 3947). 2

Table S2. Spearman's correlation (rho) among potential confounders (n= 3947). 3

Table S3. Associations of underlying diseases with healthy eating behavior and physical activity among outpatients using multiple logistic regression analysis (N = 3947). 4

Table S1. Confounders associated with suspected COVID-19 symptoms among outpatients (n= 3947).

Variables	Bivariate model	
	OR (95% CI)	*p* Value
Age groups		
18-59	1.00	
60-85	2.06 (1.77, 2.40)	<0.001
Gender		
Female	1.00	
Male	1.02 (0.89, 1.16)	0.762
Marital status		
Single	1.00	
Married	1.81 (1.52, 2.15)	<0.001
Separated/Divorced/Widowed	3.37 (2.48, 4.58)	<0.001
Education levels		
Junior high school or below	1.00	
Senior high school	0.61 (0.52, 0.73)	<0.001
College/university/postgraduate degree	0.73 (0.63, 0.85)	<0.001
Occupational status		
No job	1.00	
Have a job	1.19 (1.04, 1.36)	0.010
Ability to pay for healthcare		
Very or fairly difficult	1.00	
Very or fairly easy	0.44 (0.39, 0.51)	<0.001
Social status		
Low	1.00	
Middle or high	0.62 (0.51, 0.75)	<0.001
BMI, kg/m2		
Underweight (BMI < 18.5)	1.28 (1.03, 1.59)	0.024
Normal weight (18.5 ≤ BMI < 25.0)	1.00	
Overweight/obese (BMI ≥ 25.0)	0.61 (0.48, 0.77)	<0.001
Underlying diseases		
No	1.00	
Yes	0.99 (0.83, 1.19)	0.936
Smoking		
No	1.00	
Yes	1.06 (0.86, 1.29)	0.580
Drinking alcohol		
No	1.00	
Yes	0.90 (0.78, 1.04)	0.140
Health literacy, 1-score increment	0.95 (0.94, 0.96)	<0.001

Abbreviations: OR, odds ratio; BMI, Body Mass Index.

Table S2. Spearman's correlation (rho) among potential confounders (n= 3947).

Variables	Age	Gender	Marital status	Education	Occupation	Ability to pay	Social status	Underlying diseases	BMI	Smoking	Drinking	HES	Physical activity
Gender	-.00												
Marital status	.31	-.11											
Education	-.29	.07	**-.32**										
Occupation	-.23	.13	.07	.07									
Ability to pay	-.16	-.01	-.11	.15	.06								
Social status	-.15	.04	-.08	.19	.02	.24							
Underlying diseases	.29	-.01	.15	-.14	-.13	-.05	-.03						
BMI	.04	.07	0.1	-.00	.04	.02	.06	.06					
Smoking	-.01	**.35**	.02	-.07	.13	-.04	-.00	-.00	.07				
Drinking	-.11	**.44**	-.09	.11	.14	.01	.02	-.04	.04	**.36**			
HES	.00	-.03	-.04	.09	-.04	.07	.02	.00	-.02	-.09	-.11		
Physical activity	-.19	.03	-.08	.12	.04	.07	.04	-.07	-.01	-.03	.05	.05	
Health literacy	-.29	.08	-.24	**.32**	.06	.26	.24	-.15	.00	-.00	.04	.07	.08

Abbreviations: HES, healthy eating score; BMI, Body Mass Index.

Table S3. Associations of underlying diseases with healthy eating behavior and physical activity among outpatients using multiple logistic regression analysis (N = 3947).

Variables	Healthy Eating Behavior *				Physical activity**			
	OR (95% CI)	*p*	OR (95% CI)	*p*	OR (95% CI)	*p*		
**Underlying diseases**								
**No**	Ref.		**Ref.**		Ref.		**Ref.**	
**Yes**	1.17 (0.95, 1.45)	0.129	**1.14 (0.91, 1.42)**	**0.251**	0.67 (0.56, 0.81)	<0.001	**0.94 (0.76, 1.14)**	**0.516**
Age groups								
18-59	Ref.		Ref.		Ref.		Ref.	
60-85	1.19 (0.99, 1.43)	0.052	1.21 (0.99, 1.50)	0.066	0.40 (0.34, 0.47)	<0.001	0.48 (0.40, 0.57)	<0.001
Gender								
Female	Ref.		Ref.		Ref.		Ref.	
Male	1.00 (0.86, 1.17)	0.966	1.01 (0.85, 1.19)	0.926	1.17 (1.02, 1.35)	0.027	1.11 (0.94, 1.30)	0.223
Marital status								
Single	Ref.		-	-	Ref.		-	-
Married	0.81 (0.68, 0.97)	0.025	-	-	0.77 (0.64, 0.91)	0.003	-	-
Separated/Divorced/Widowed	1.46 (1.01, 2.10)	0.044	-	-	0.43 (0.32, 0.59)	<0.001	-	-
Education levels								
Junior high school or below	Ref.		Ref.		Ref.		Ref.	
Senior high school	1.05 (0.85, 1.29)	0.636	1.18 (0.95, 1.47)	0.128	1.54 (1.28, 1.83)	<0.001	1.20 (0.99, 1.45)	0.062
College/university/postgraduate degree	1.46 (1.21, 1.75)	<0.001	1.73 (1.41, 2.12)	<0.001	2.01 (1.71, 2.38)	<0.001	1.47 (1.22, 1.76)	<0.001
Occupational status								
No job	Ref.		Ref.		Ref.		Ref.	
Have a job	0.81 (0.69, 0.95)	0.008	0.84 (0.71, 0.99)	0.037	1.19 (1.04, 1.37)	0.014	0.96 (0.83, 1.12)	0.626
Ability to pay for healthcare								
Very or fairly difficult	Ref.		Ref.		Ref.		Ref.	
Very or fairly easy	0.86 (0.74, 1.01)	0.057	0.90 (0.77, 1.06)	0.221	1.46 (1.27, 1.68)	<0.001	1.25 (1.07, 1.45)	0.004
Social status								
Low	Ref.		Ref.		Ref.		Ref.	
Middle or high	0.74 (0.58, 0.94)	0.013	0.71 (0.55, 0.91)	0.007	1.43 (1.17, 1.75)	0.001	1.05 (0.84, 1.30)	0.683

BMI, kg/m2								
Underweight (BMI < 18.5)	1.19 (0.92, 1.53)	0.178	1.19 (0.92, 1.55)	0.176	0.99 (0.78, 1.25)	0.935	0.98 (0.76, 1.25)	0.86
Normal weight (18.5 ≤ BMI < 25.0)	Ref.		Ref.		Ref.		Ref.	
Overweight/obese (BMI ≥ 25.0)	1.12 (0.87, 1.43)	0.384	1.14 (0.88, 1.46)	0.329	0.85 (0.68, 1.06)	0.156	0.88 (0.69, 1.10)	0.266
Smoking								
No	Ref.		Ref.		Ref.		-	-
Yes	0.81 (0.63, 1.05)	0.116	0.89 (0.68, 1.19)	0.446	0.85 (0.69, 1.05)	0.140	-	-
Drinking alcohol								
No	Ref.		-	-	Ref.		Ref.	
Yes	0.91 (0.77, 1.08)	0.271	-	-	1.32 (1.14, 1.54)	<0.001	1.12 (0.94, 1.34)	0.220
Health literacy, 1-score increment	1.01 (0.99, 1.02)	0.183	-	-	1.03 (1.02, 1.04)	<0.001	-	-

* The reference group is 'low HES', the test group is 'medium or high HES'.

** The reference group is 'physical inactivity', the test group is 'active physical activity'.
